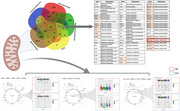# Integrative Systems Approaches Identifying Mitochondrial Metabolic Disruptions in Alzheimer's Disease

**DOI:** 10.1002/alz70856_106631

**Published:** 2026-01-09

**Authors:** Priyanka Baloni, Matthias Arnold, Jesse C Wiley, Huanyao Gao, Cory C Funk, Richa Batra, Alexandra Kueider‐Paisley, Bin Zhang, Russell H Swerdlow, Eugenia Trushina, Rima F. Kaddurah‐Daouk, Alzheimer Disease Metabolomics Consortium

**Affiliations:** ^1^ Purdue University, West Lafayette, IN, USA; ^2^ Duke University, Durham, NC, USA; ^3^ Department of Psychiatry and Behavioral Sciences, Duke University, Durham, NC, USA; ^4^ German Center for Diabetes Research (DZD), Neuherberg, Germany; ^5^ Helmholtz Zentrum München ‐ German Research Center for Environmental Health, Neuherberg, 85764, Germany; ^6^ University of Washington, Seattle, WA, USA; ^7^ Sage Bionetworks, Seattle, WA, USA; ^8^ Yale University, New Haven, CT, USA; ^9^ Institute for Systems Biology, Seattle, WA, USA; ^10^ Institute for Computational Biomedicine, Englander Institute for Precision Medicine, Department of Physiology and Biophysics, Weill Cornell Medicine, New York, NY, USA; ^11^ Icahn Genomics Institute, Icahn School of Medicine at Mount Sinai, New York, NY, USA; ^12^ University of Kansas Alzheimer's Disease Research Center, Fairway, KS, USA; ^13^ Mayo Clinic, Rochester, MN, USA; ^14^ Duke University Medical Center, Durham, NC, USA

## Abstract

**Background:**

Mitochondrial dysfunction in Alzheimer's Disease (AD) is characterized by impaired energy production, oxidative stress, and disrupted calcium homeostasis, which collectively contribute to neuronal damage, synaptic dysfunction, and the progression of AD pathology. A significant knowledge gap in studying mitochondrial dysfunction in Alzheimer's Disease (AD) lies in understanding the precise molecular mechanisms linking mitochondrial metabolic alterations to neuronal damage, particularly how sex‐specific and cell‐type‐specific mitochondrial processes contribute to the progression of AD pathology.

**Method:**

This study integrates multiple datasets, post‐mortem brain RNAseq from ROSMAP, Mayo Clinic brain bank and Mount Sinai Brain Bank cohort data available via AD Knowledge Portal hosted by Sage Bionetworks), Human Protein Atlas (HPA), MitoCarta3, genome‐scale human metabolic reconstruction, mitochondriome analysis, and brain & blood metabolomics data (data generated by the Alzheimer's Disease Metabolomics Consortium (ADMC)) to identify mitochondrial genes associated with disrupted metabolism in AD. Additionally, biodomain analysis was used to identify key processes associated with pathology.

**Result:**

A total of 62 common genes were identified across these approaches, highlighting their potential significance in mitochondrial processes. We also developed the framework to perform metabolite‐gene correlation analyses revealing key mitochondrial‐metabolic interactions (eg. homocysteine and MTHFR gene; acetyl‐CoA and PDHA1 gene). Integrated in silico metabolic analysis identified reaction fluxes, uncovering sex‐specific differences in mitochondrial transport and tricarboxylic acid (TCA) cycle subsystems, with notable alterations in females. Biodomain enrichment analysis focused on mitochondrial processes, identifying disruptions in pathways critical for energy production and redox homeostasis. Additionally, we analyzed datasets related to metformin and complex I inhibitors, linking mitochondrial complex I modulation with metabolic alterations in AD. This analysis identified druggable targets and potential therapeutic candidates for mitochondrial metabolic pathways.

**Conclusion:**

Our findings integrate multiple data sources to provide a systems‐level understanding of mitochondrial metabolism in AD. Sex‐specific differences associated with mitochondrial dysfunction in AD were identified. We identify important mitochondrial transport proteins and metabolites that could potentially be used as druggable targets in AD.